# Ultrasound Diagnosis of Subhepatic Appendicitis

**DOI:** 10.3390/reports9040319

**Published:** 2026-09-22

**Authors:** Dragan Vasin, Djuro Cutura, Djordjije Saranovic

**Affiliations:** 1 Emergency Radiology Department, Center for Radiology, University Clinical Center of Serbia, Pasterova Str. No 2, 11000 Belgrade, Serbia; 2 General Hospital Medigroup, Milutina Milankovica 3, 11000 Belgrade, Serbia

**Keywords:** appendicitis, subhepatic, ultrasound, technique

## Abstract

Acute subhepatic appendicitis is an exceptionally rare anatomical variant (accounting for 0.01–0.09% of all appendicitis cases) that arises from embryological intestinal malrotation or incomplete cecal descent. A 39-year-old male presented to the emergency department with a 48-h history of progressive right upper quadrant pain, anorexia, mild nausea, low-grade fever (37.8 °C), marked leukocytosis (19 × 10^9^/L with a left shift), and elevated C-reactive protein (5.2 mg/L). Physical examination demonstrated localized tenderness and guarding in the right hypochondrium, with a negative clinical Murphy’s sign. Bedside abdominal ultrasonography was performed utilizing a dedicated dual-probe strategy: a low-frequency convex transducer (1–5 MHz) for anatomical orientation and a high-frequency linear transducer (6–12 MHz) for targeted near-field interrogation. Graded compression identified a non-compressible, aperistaltic, blind-ending tubular structure in the hepatorenal recess (Morison’s pouch) measuring 9 mm in outer diameter with a thickened wall (3.2 mm), an appendicolith at the base, mural hyperemia on color Doppler, and surrounding hyperechoic fat stranding with localized free fluid. Real-time sonography systematically excluded acute cholecystitis, hepatic lesions, and perforated peptic ulcer. Preoperative CT was omitted, and the patient proceeded directly to emergent diagnostic laparoscopy, which confirmed an inflamed, non-perforated appendix in the right subhepatic space adjacent to the liver margin. A laparoscopic appendectomy was successfully performed. Histopathology confirmed uncomplicated suppurative appendicitis, and the patient was discharged uneventfully on postoperative day three. Emergency high-resolution ultrasonography using a targeted dual-probe technique can conclusively diagnose subhepatic appendicitis, challenging the paradigm that CT is strictly mandatory. In patients presenting with atypical right upper quadrant pain, dedicated sonographic interrogation of the hepatorenal space facilitates prompt surgical management while eliminating ionizing radiation and diagnostic delays.

A 39-year-old man presented to the emergency department with a 48-h history of acute, progressive, and non-radiating pain in the right hypochondrium, accompanied by anorexia and mild nausea, without vomiting, jaundice, or altered bowel habits. On admission, he was subfebrile with a body temperature of 37.8 °C. Physical examination revealed localized tenderness and mild voluntary guarding in the right upper quadrant; clinical Murphy’s sign, rebound tenderness, and signs of generalized peritonitis were absent. Laboratory evaluation demonstrated marked leukocytosis (white blood cell count: 19 × 10^9^/L with a left shift) and a mildly elevated C-reactive protein level of 5.2 mg/L. A bedside abdominal ultrasound was performed using a high-resolution ultrasound system (GE Logiq E9), equipped with both a low-frequency convex probe (1–5 MHz) for deep anatomical orientation and a high-frequency linear transducer (6–12 MHz) for detailed superficial and near-field assessment. Scanning was performed via targeted transabdominal subcostal and lateral intercostal approaches, focused on the hepatorenal recess (Morrison’s pouch).

Utilizing the graded compression technique, the examination revealed a blind-ending, non-compressible, tubular, aperistaltic structure located in the hepatorenal space between the right kidney and liver, corresponding precisely to the point of maximal sonographic tenderness (Figure 1, Figure 2 and Figure 3). The structure demonstrated an increased outer transverse diameter of 9 mm with circumferential wall thickening of 3.2 mm. Color Doppler interrogation revealed marked mural hyperemia consistent with active inflammation. In addition, prominent, hyperechoic, non-compressible, periappendiceal fat changes (inflammatory fat stranding) and a small amount of localized free fluid within the hepatorenal pouch were clearly identified (Figure 1, Figure 2 and Figure 3). Diagnostic laparoscopy substantiated the preoperative ultrasound diagnosis of acute appendicitis occurring within the context of subhepatic localization. Intraoperatively, the abnormally positioned cecum and an inflamed, indurated appendix were encountered in the right subhepatic space, close to the duodenum and liver edge, fully corresponding to the nonrotation/malrotation pattern and localized inflammatory changes seen on imaging. A laparoscopic appendectomy was successfully performed (Figure 4). The appendix did not have any perforations, and the patient was discharged on the third day after the intervention without any complications. The postoperative histopathological examination confirmed acute simple (uncomplicated) appendicitis (phlegmonous/suppurative inflammation confined to the mucosal and muscular layers), with no evidence of transmural necrosis, gangrene, perforation, or periappendicular abscess formation.

Appendicitis is the most common surgical emergency and the leading cause of acute abdomen, and awareness of anatomical variants of the appendix position is necessary in order to correctly diagnose and safely manage appendicitis. Subhepatic acute appendicitis is extremely rare, accounting for approximately 0.01% to 0.09% of all acute appendicitis presentations. It is typically the result of embryological variations, such as incomplete intestinal rotation or failure of the cecum to descend into the right iliac fossa during fetal development.

For subhepatic appendicitis, ultrasonography (US) can be a practical bedside imaging method with a high-frequency probe (HFP) for the colon and liver. Establishing a definitive diagnosis of subhepatic appendicitis via US is exceptionally uncommon due to several anatomical and technical factors, including acoustic shadowing from bowel gas, failure of graded compression, and structural adherence; thus, the resulting periappendiceal inflammation and edema, such as pericholecystic fluid, gallbladder wall thickening, or atypical liver abscesses, are frequently misidentified on ultrasound. What makes this case unique compared with the previously reported literature is that a conclusive diagnosis was achieved exclusively using emergency abdominal ultrasonography, avoiding the delays, costs, and ionizing radiation associated with preoperative CT. This was made possible by a targeted dual-probe scanning strategy. The diagnosis of subhepatic appendicitis is almost always established by computed tomography (CT) [1]. While CT is widely regarded as the gold standard, pre-operative CT was omitted in our patient. Initial emergency abdominal ultrasonography conclusively identified the atypically positioned, non-compressible, and inflamed subhepatic tubular structure with surrounding inflammatory changes, which—alongside marked localized peritonism—provided sufficient diagnostic confidence to proceed directly to surgery without procedural delay or radiation exposure. In our view, specifically in subhepatic appendicitis, the additional advantages of HFP include good contact with the body surface and a good acoustic window from the liver surface, which is why we found the high-frequency linear probe, transversely at the liver surface, effective in detecting the subhepatic position of the appendix and periappendiceal free fluids [2].

Several conditions mimic and may be confused with subhepatically located appendicitis. A critical diagnostic challenge in this anatomical location is distinguishing appendicitis from other acute conditions of the right upper quadrant. In our patient, alternative diagnoses were systematically excluded using real-time sonography:

- Acute cholecystitis was ruled out by identifying a normally distended thin-walled (<3 mm) gallbladder containing clear anechoic bile without calculi, sludge, or pericholecystic fluid halo, alongside a negative sonographic Murphy’s sign.
- A hepatic or subhepatic abscess was excluded by demonstrating a homogeneous hepatic parenchymal echotexture without focal intrahepatic hypo- or heterogeneous lesions, liquefactive necrosis, or peripheral wall hypervascularity; the inflammatory changes were strictly extrahepatic and contiguous with the tubular intestinal structure.
- A perforated peptic or duodenal ulcer was excluded by the absence of indirect sonographic signs of pneumoperitoneum (such as peritoneal stripe enhancement or reverberation/comet-tail artifacts anterior to the liver), no focal duodenal mural disruption, and no loculated extraluminal fluid collections containing echogenic microbubbles in Morison’s pouch.
- Other potential mimics, including cecal or ascending colon diverticulitis, foramen of Winslow internal herniation, and necrotic subhepatic lymphadenopathy, were discounted by tracing the continuous blind-ended tubular morphology back to the cecal base [3].

Subhepatic appendicitis has several associated adverse outcomes, including a delay in patient treatment, possible mucocele formation in uncomplicated appendicitis, and an increased risk of complications such as subphrenic abscess formation, right pleural effusion, and right posterobasal pneumonia. In the case of a clinical suspicion of appendicitis not accompanied by positivity upon conventional examination, the routine use of subhepatic scans should be considered as the first choice in emergency rooms, especially in the pediatric population, for confirmation of undiagnosed subhepatic appendicitis [4]. Although contrast-enhanced computed tomography (CT) remains the definitive imaging modality of choice for confirming ectopic appendicitis and assessing complications, a targeted high-resolution ultrasound examination of the right upper quadrant (RUQ) can serve as a valuable initial radiation-free tool. In experienced hands and when clinical suspicion arises, graded compression of the point of maximum tenderness may identify subhepatic appendicitis at an early uncomplicated stage, while simultaneously ruling out more common acute hepatobiliary pathologies. This prompt sonographic identification prevents diagnostic delays, averts severe local complications, and directly enables a safe, uncomplicated, and successful laparoscopic appendectomy.

In patients presenting with atypical RUQ pain mimicking acute cholecystitis, subhepatic appendicitis should be considered in the differential diagnosis. While ultrasound serves as an accessible first-line assessment to rule out hepatobiliary disease, contrast-enhanced CT remains essential for a definitive diagnosis and treatment planning when sonographic findings are equivocal.

## Figures and Tables

**Figure 1:**
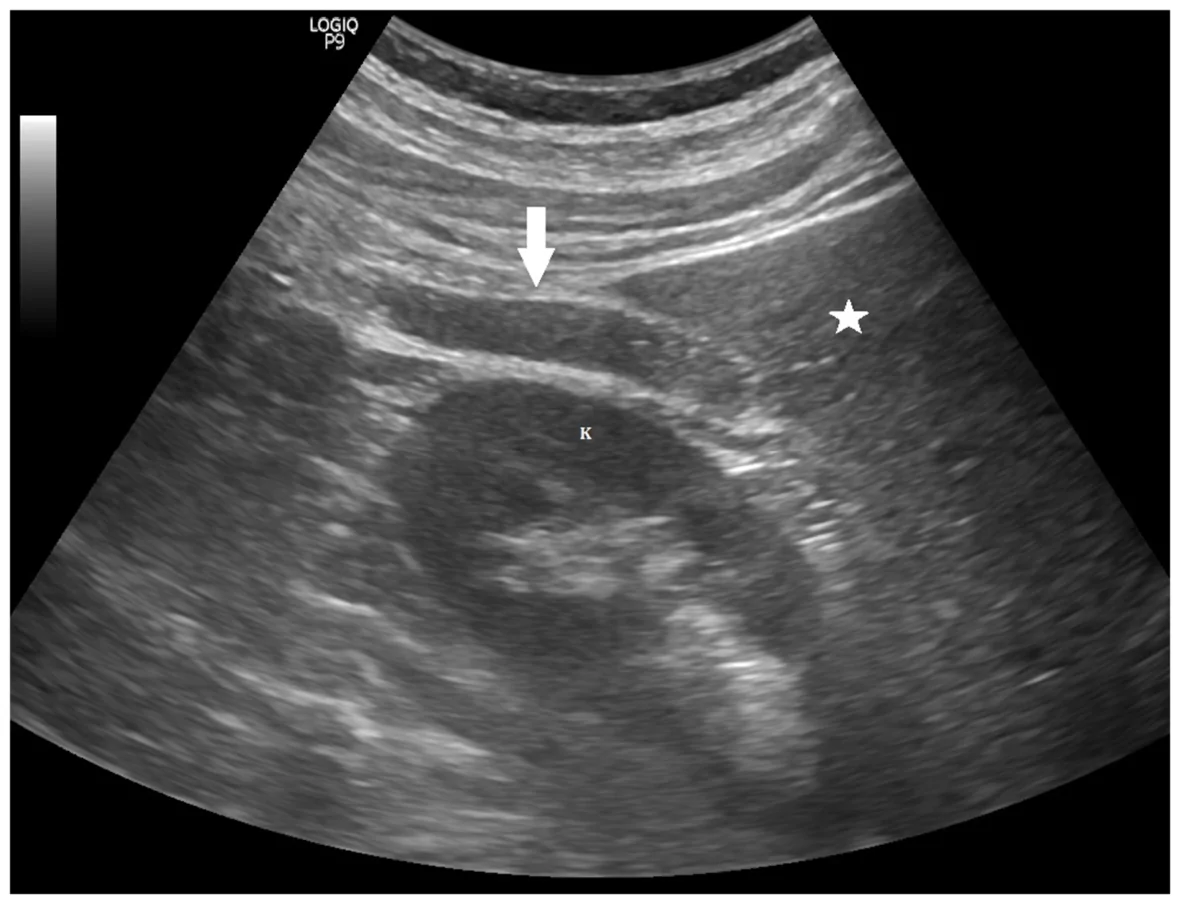
The ultrasound oblique scan in the lateral subhepatic region (white star: liver; K: kidney) revealed a non-compressible, blind-ended, distended, aperistaltic hypoechoic tubular structure (white arrow) with a “target” appearance, showing positive probe tenderness on compression and an outer transverse diameter of 9 mm, which are ultrasound signs of subhepatic appendicitis.

**Figure 2:**
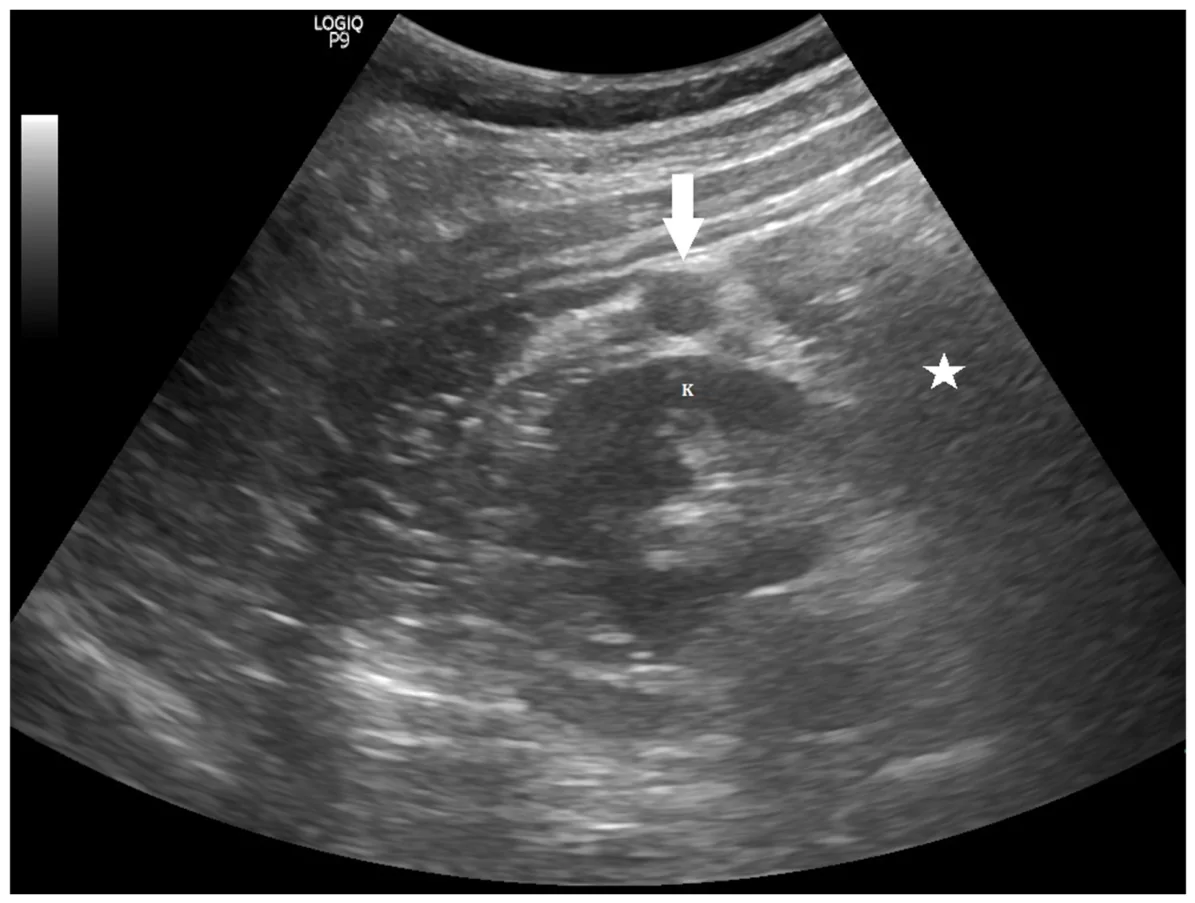
The ultrasound transverse scan in the lateral subhepatic region (white star: liver; K: kidney) revealed a non-compressible, distended, aperistaltic hypoechoic structure (white arrow) with a “target” appearance, an outer transverse diameter of 9 mm, and an inflamed mesoappendix, which are ultrasound signs of subhepatic appendicitis.

**Figure 3:**
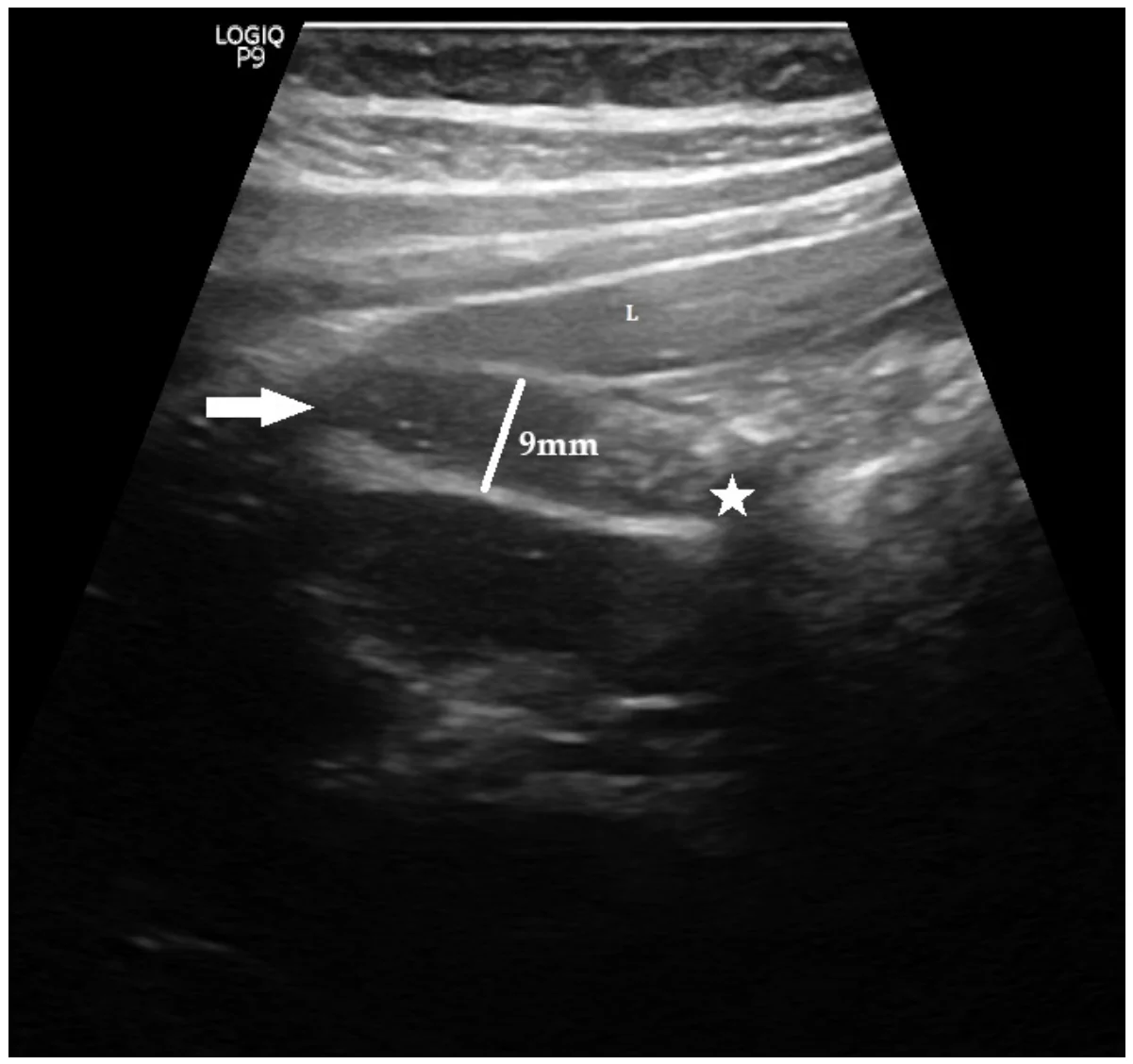
High-frequency ultrasound showing subhepatic appendicitis (white arrow; L: liver) with an appendicolith at its appendiceal base (white star).

**Figure 4:**
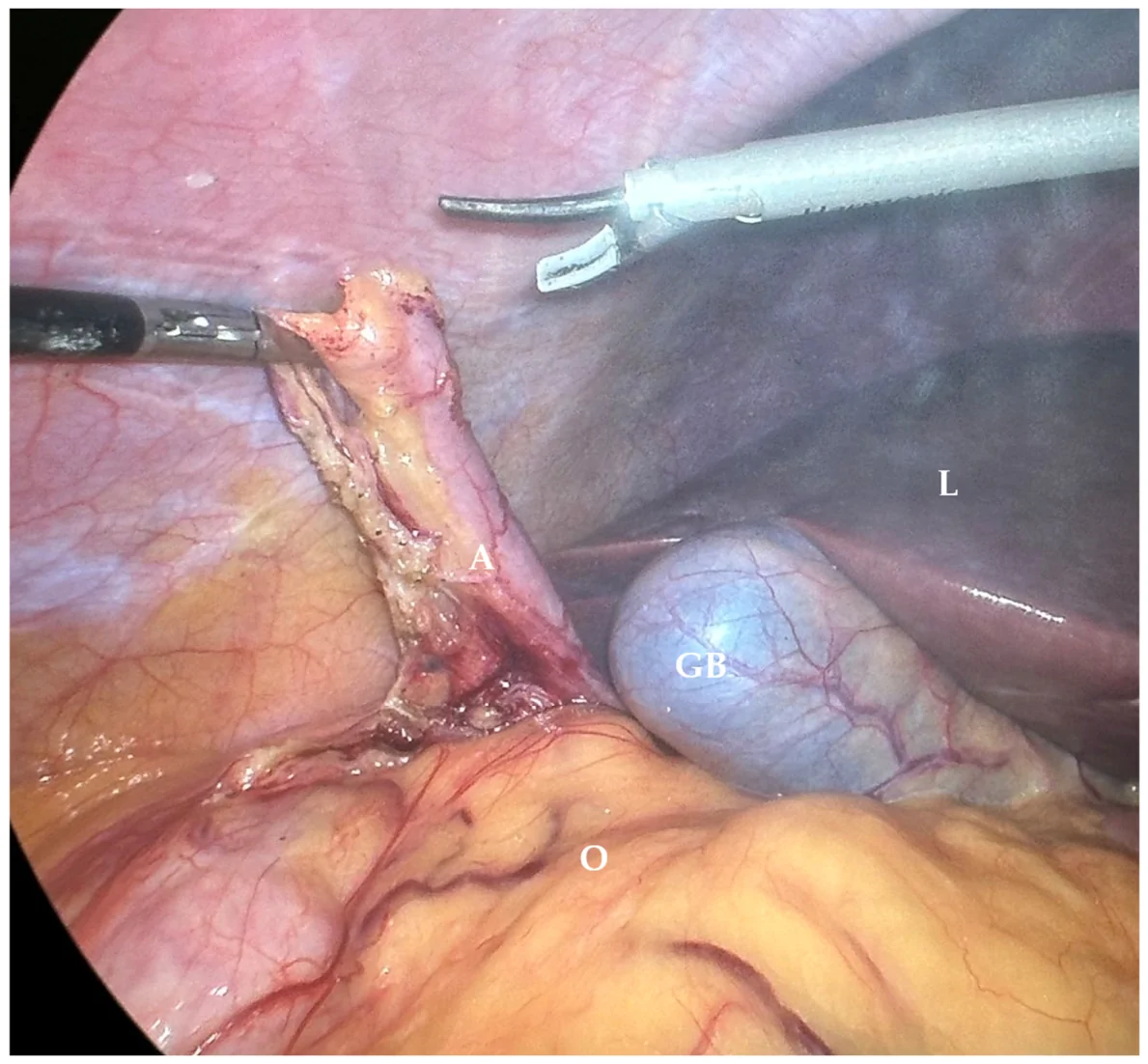
Intraoperative laparoscopic view confirming the preoperative imaging findings: an inflamed, thickened appendix located in the right subhepatic space, secondary to the right liver lobe edge (GB: gallbladder; L: liver; O: omentum).

## Data Availability

The data underlying this study are included in the article; further inquiries can be directed to the corresponding author.
